# First principle investigation of Mo substitution on the structural mechanical and thermal stability of TiMoB_2_ solid solutions

**DOI:** 10.1038/s41598-025-91604-w

**Published:** 2025-04-13

**Authors:** Wondayehu Yeshewas Alemu, Cheng-Yen Lee, Hsin-An Chen, Jhewn-Kuang Chen

**Affiliations:** Institute of Materials Science and Engineering, National Taipei University of Technology, No.1, Sec. 3, Zhong-Xiao E. Rd, Taipei, 106344 Taiwan

**Keywords:** Ti–Mo–B_2_, Density functional theory, Mechanical properties, Thermal properties, Condensed-matter physics, Structural materials, Theory and computation, Applied physics, Condensed-matter physics

## Abstract

Titanium diboride (TiB_2_) and molybdenum diboride (MoB_2_) are known for their excellent mechanical properties such as high hardness, wear resistance and thermal stability, of great interest in advanced engineering applications. This study systematically explored the structural, electronic, thermal, and mechanical properties of Ti–Mo–B_2_ solid solutions via first-principles density functional theory (DFT). Mo is substituted into the TiB_2_ lattice to investigate its effect on five alloy compositions of key material properties. Our analysis revealed that increasing Mo content enhances ductility while reducing stiffness and hardness, transitioning from a more rigid, covalent structure in TiB_2_ to a more ductile, metallic behavior in MoB_2_, as shown by the rise in Poisson’s ratio from 0.13 in TiB_2_ to 0.26 in MoB_2_ and the Pugh’s ratio increase from 1.00 to 1.70. Mo substitution reduces Debye temperature as well as melting points. Phonon dispersion calculations show that the Ti_0.5_Mo_0.5_B_2_ solid solution exhibits dynamical stability, making it a promising composition for enhanced mechanical and thermal stability. Our studies also demonstrate that the alloys form stable solid solutions across all compositions, with stability reflected by negative mixing energies. These findings provide a key information into designing high-performance Ti–Mo–B_2_ composites with specific mechanical and thermal characteristics.

## Introduction

TiB_2_ with its hexagonal crystal structure (P6/mmm No. 191) is a great material for advanced engineering applications due to its high hardness, good wear resistance and thermal stability. It is good for cutting tools, protective coatings and armor materials^1–3^. Also, it is used in electrochemical reduction of alumina to aluminum^4^. But its brittleness limits its wider use especially in applications that requires both toughness and hardness^5^.

One of a promising solution to TiB_2_’s brittleness is to alloy it with transition metal borides like molybdenum diboride (MoB_2_). Molybdenum with its larger atomic size and different electronic configuration introduces new atomic interactions when substituted into the TiB_2_ lattice and modify the mechanical and electronic properties^6^. Mo substitution is expected to improve toughness while retaining the hardness and thermal stability of TiB_2_ and hence Ti–Mo–B_2_ alloys are suitable for high performance applications. Understanding these atomic level changes is important to design alloys that balance hardness and toughness, key to develop more durable materials^7–9^.

Although intensive study has been performed on pure TiB_2_ and MoB_2_, the investigations of its alloy are rare especially in their electronic properties and mechanical properties. This gap in knowledge presents an opportunity for further investigation into Ti–Mo–B_2_ solid solutions, particularly given that previous studies on other solid solutions, like ZrC–ZrN and TiC–TiN have indicated that optimal mechanical properties often occur at intermediate compositions^10,11^. While these findings are well established in carbide and nitride systems, their implications for boride-based solid solutions remain largely unexplored. A previous computational study on transition metal diborides (TMB_2_) have showed the role of alloying and defect engineering in enhancing phase stability and mechanical properties. For instance, research on W-based diborides shows that metastable α-phase structures can achieve greater ductility when stabilized by alloying elements like Ta or through vacancy control. Applying similar strategies to Ti–Mo–B_2_ alloys could help optimize the balance between hardness and toughness, further advancing the development of high-performance boride-based materials^12^. Inspired by these findings, we hypothesize that comparable behavior might be seen also in the Ti–Mo–B_2_ alloys due to the influence of electronic structure on strength enhancements^13,14^. Despite these advances in other material systems, the specific effects of Mo substitution in TiB_2_ remain largely unknown. In particular, little is understood about how Mo incorporation influences the electronic density of states, bonding characteristics, and mechanical stability of TiB_2_-based solid solutions.

In light of this work, the study aims to fill some of these gaps in the understanding of Ti–Mo–B_2_ solid solutions through a detailed analysis that can be benchmarked on their structural, electronic, mechanical as well as thermal properties using first principles calculations. We studied different properties such as the total energy, the density of states (DOS), elastic constants, hardness, Debye temperature and melting temperature with respect to composition. It will be useful for application of TiB_2_ based alloys designing for high performance industrial applications.

## Results and discussion

### Structural properties and stability of the alloy

**Fig. 1:**
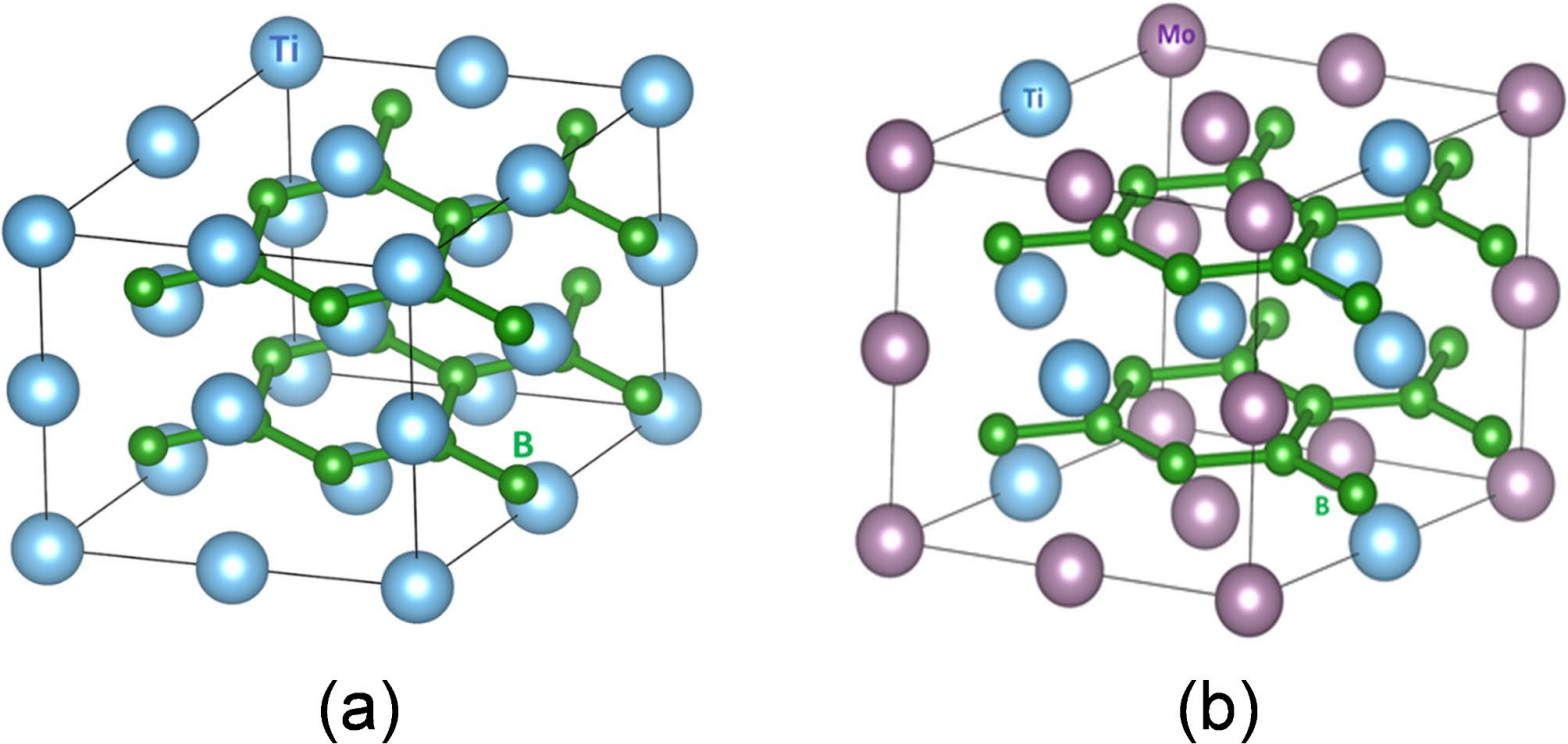
Structural configurations of the metal atoms in Ti_1–x_Mo_x_B_2_(x = (**a**) 0.0, and (**b**) 0.5).

**Fig. 2:**
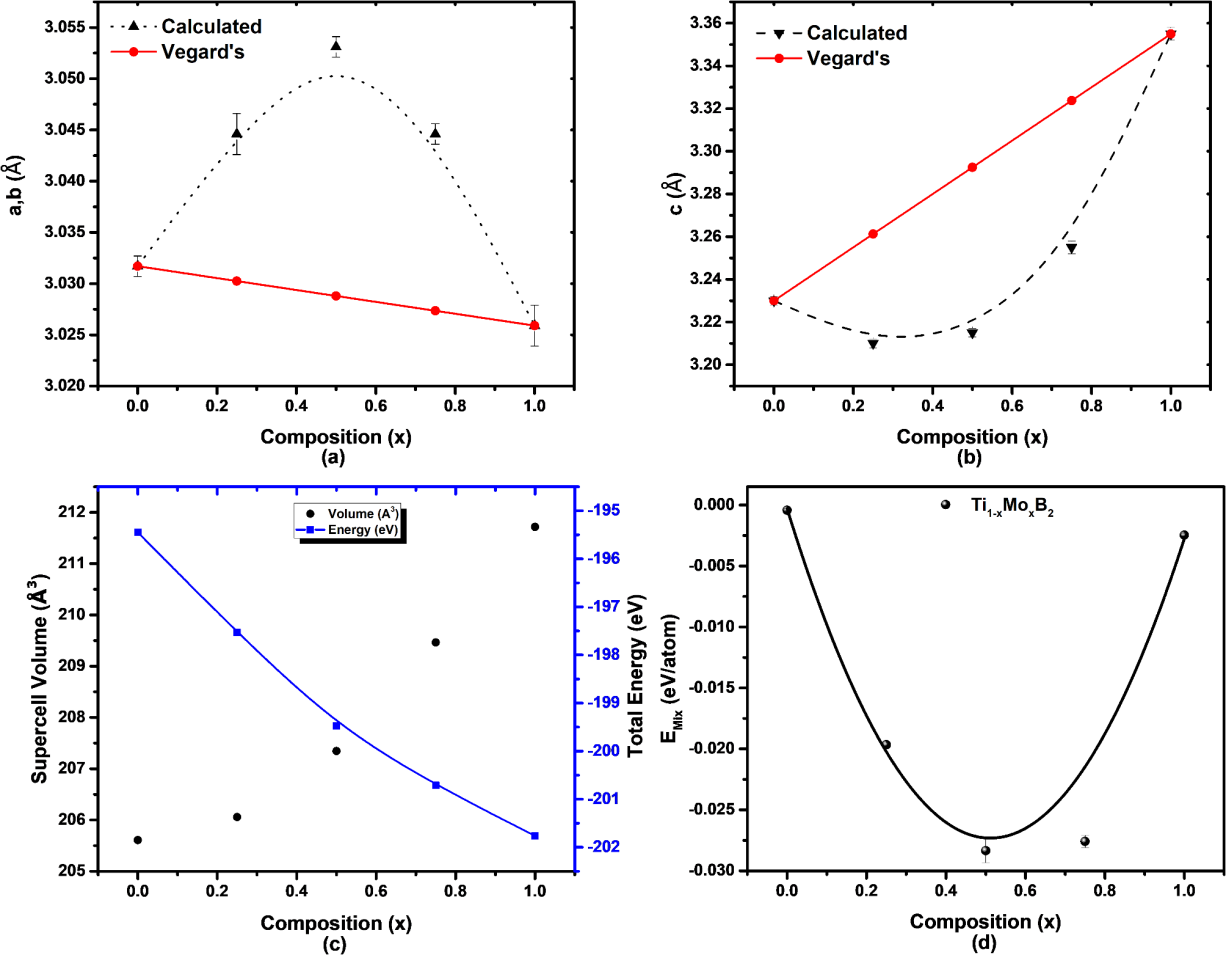
Lattice parameters of (**a**) a, b, (**b**) c, (**c**) volume versus total energy and (**d**) energy of mixing versus composition of Ti_1-x_Mo_x_B_2_ (The lines are the resulting polynomial fit to the calculated points).

Figure 1 shows the atomic structure configurations of Ti_1–x_Mo_x_B_2_ alloys (x = 0.0 and 0.5) for a 2 × 2 × 2 supercell. Where Ti (Mo) atoms are represented by large blue (Brown) spheres and B atoms are shown as small green spheres. For x = 0.0, the structure is pure TiB_2_, with a uniform distribution of titanium and boron atoms. As Mo atoms substitute half of the Ti atoms (x = 0.5), the differing atomic radii and bonding characteristics of Mo introduce lattice distortions. These distortions increase the lattice volume due to Mo’s larger atomic size, impacting both the local structure and overall stability of the alloy, as seen in the structural optimization and total energy calculations (Fig. 2c). The increase in volume with Mo content aligns with Mo’s larger atomic radius compared to Ti. And also, the reduction in total energy with higher Mo concentrations suggests structural stability, likely due to lattice relaxation and reduced internal strain.

Figure 2a,b presents the lattice parameters a and c for TiB_2_-MoB_2_ alloys, obtained via VASP structural optimization as a function of composition. While the supercell volume increases linearly with Mo content, neither lattice parameter a nor c follows Vegard’s Law^15^. The lattice parameter c appears to have quite a consistent negative deviation from linear prediction while the lattice parameter a displays positive deviation from the prediction. These findings indicate that intra-layer atomic interactions are stronger in the c direction than the a and b directions. This trend corresponds with previous findings on comparable Ti-Nb-B_2_ based alloys^16^. The positive deviations from Vegard’s Law for ‘a’ and negative for ‘c’ mark the limitations of a simple linear approximation in predicting the behavior of alloys. These deviations are due to the heterogeneity in atomic size, shape, coordination and bonding.

Our calculated lattice parameters for TiB_2_ (a = 3.0317 Å, c = 3.2289 Å) and MoB_2_ ( a = 3.0259, c = 3.3375 Å) match well with experimental^17,18^ and most theoretical^19,20^ values from other studies. Additionally, our calculations reveal that the Ti-B bond lengths are approximately 2.380 Å and the B-B bond lengths are around 1.751 Å. In contrast, Mo-B bonds are slightly longer at approximately 2.416 Å, with B-B bond lengths measuring about 1.747 Å. The shorter Ti-B bond may enhance the bonding strength and increases the hardness of TiB_2_ compared to MoB_2_, which demonstrates slightly lower elasticity due to its longer bond length.

The stability of Ti_1–x_Mo_x_B_2_ alloys is fundamentally determined by the Gibbs free energy of mixing at zero Kelvin. At this temperature, both entropy and phonon contributions are negligible, simplifying the stability analysis to a focus on the mixing energy, which can be expressed as:

1 $${\text{E}}_{{{\text{Mix}}}} \left( {\text{x}} \right) = {\text{E}}_{{\text{T}}} \left( {{\text{Ti}}_{{1 - {\text{x}}}} {\text{Mo}}_{{\text{x}}} {\text{B}}_{2} } \right) - {\text{x}} \times {\text{E}}_{{\text{T}}} \left( {{\text{MoB}}_{2} } \right) - \left( {1 - {\text{x}}} \right) \times {\text{E}}_{{\text{T}}} \left( {{\text{TiB}}_{2} } \right)$$

where E_T_ represents the total energies of the alloy, MoB_2_, and TiB_2_, respectively. The calculated mixing energies, as shown in Fig. 2d, indicate that it is energetically favorable for MoB_2_ and TiB_2_ to mix and form alloys across the entire composition range. A polynomial fit to the mixing energy data reveals that the minimum energy occurs at *x* = 0.45, suggesting the most stable alloy composition.

### Electronic properties and boning nature

Bader^21^ charge analysis gives important information on the electronic charge distribution and atomic charges. It clearly elucidated the topology of electron density in every atomic basin, offering insight into how electrons are distributed and shared among atoms. This method captures both charge accumulation, indicative of covalent bonding, and charge depletion, representative of ionic character. It provides the charge transfer and nature of bonding in TiB_2_, MoB_2_, and Ti_0.5_Mo_0.5_B_2_ alloys.

From Table 1, it is observed that titanium in TiB_2_ retains an average of 11.10 electrons, meaning thereby it has lost about 0.90 electrons, experiences partial electron delocalization, contributing to its metallic nature, while boron has 3.45 electrons points to strong covalent interactions in the B-B bonds within the boron sublattice (also indicated in ELF). This balance between covalent and ionic interactions results in the characteristic high hardness and thermal stability of TiB_2_.

In contrast, MoB_2_ exhibits increased metallicity, as demonstrated by molybdenum’s higher charge retention of 13.25 electrons compared to titanium. The slightly lower boron charge retention of 3.38 electrons reflects greater electron sharing, indicative of enhanced metallic bonding. This suggests that MoB_2_ might possess a softer and more ductile nature compared to TiB_2_, consistent with its lower hardness but higher electrical conductivity.

For the Ti_0.5_Mo_0.5_B_2_ alloy, the intermediate charge distribution between TiB_2_ and MoB_2_ underscores its role as a hybrid material, combining the mechanical robustness of TiB_2_ with the enhanced electrical and thermal properties of MoB_2_. The alloy’s charge distribution suggests a gradual transition in bonding characteristics, enabling a tunable balance between covalent and metallic interactions, which can be exploited for specific applications requiring tailored mechanical and electronic properties.

For Ti_0.75_Mo_0.25_B_2_, the Bader charge data shows that titanium retains 11.12 electrons, similar to pure TiB_2_, while Mo retains 13.15 electrons, slightly higher than in Ti_0.5_Mo_0.5_B_2_. Boron has a charge of 3.44 electrons, reinforcing strong B-B covalent interactions. The predominance of Ti-related charge transfer suggests that this composition retains more of the covalent and ionic character of TiB_2_ while incorporating minor metallic contributions from Mo, leading to a material with improved toughness but still maintaining high hardness. For Ti_0.25_Mo_0.75_B_2_, the trend shifts further toward MoB_2_-like behavior, with Mo retaining 13.17 electrons and Ti maintaining 11.12 electrons. The boron charge of 3.42 electrons suggests a slight weakening of covalent bonding as Mo content increases. This indicates a progressive enhancement in metallic character while still preserving some covalent bonding, which could improve electrical and thermal conductivity while reducing hardness compared to lower Mo compositions.

**Table 1:** Average bader charge differences for Ti, Mo, and B in TiB_2_, MoB_2_, Ti_0.5_Mo_0.5_B_2_, Ti_0.75_Mo_0.25_B_2_, and Ti_0.25_Mo_0.75_B_2_, highlighting charge transfer and bonding characteristics.

Compound	Element	Bader charge (final)	Charge (Initial)	Bader charge difference (ΔQ)
TiB_2_	Ti	11.10	12.0	+ 0.90
	B	3.450	3.00	− 0.45
MoB_2_	Mo	13.25	14.0	+ 0.75
	B	3.380	3.00	− 0.38
Ti_0.5_Mo_0.5_B_2_	Ti	11.05	12.0	+ 0.95
	Mo	13.21	14.0	+ 0.79
	B	3.400	3.00	− 0.40
Ti_0.75_Mo_0.25_B_2_	Ti	11.12	12.0	+ 0.88
	Mo	13.15	14.0	+ 0.85
	B	3.44	3.00	− 0.44
Ti_0.25_Mo_0.75_B_2_	Ti	11.12	12.0	+ 0.88
	Mo	13.17	14.0	+ 0.83
	B	3.42	3.00	− 0.42

**Fig. 3:**
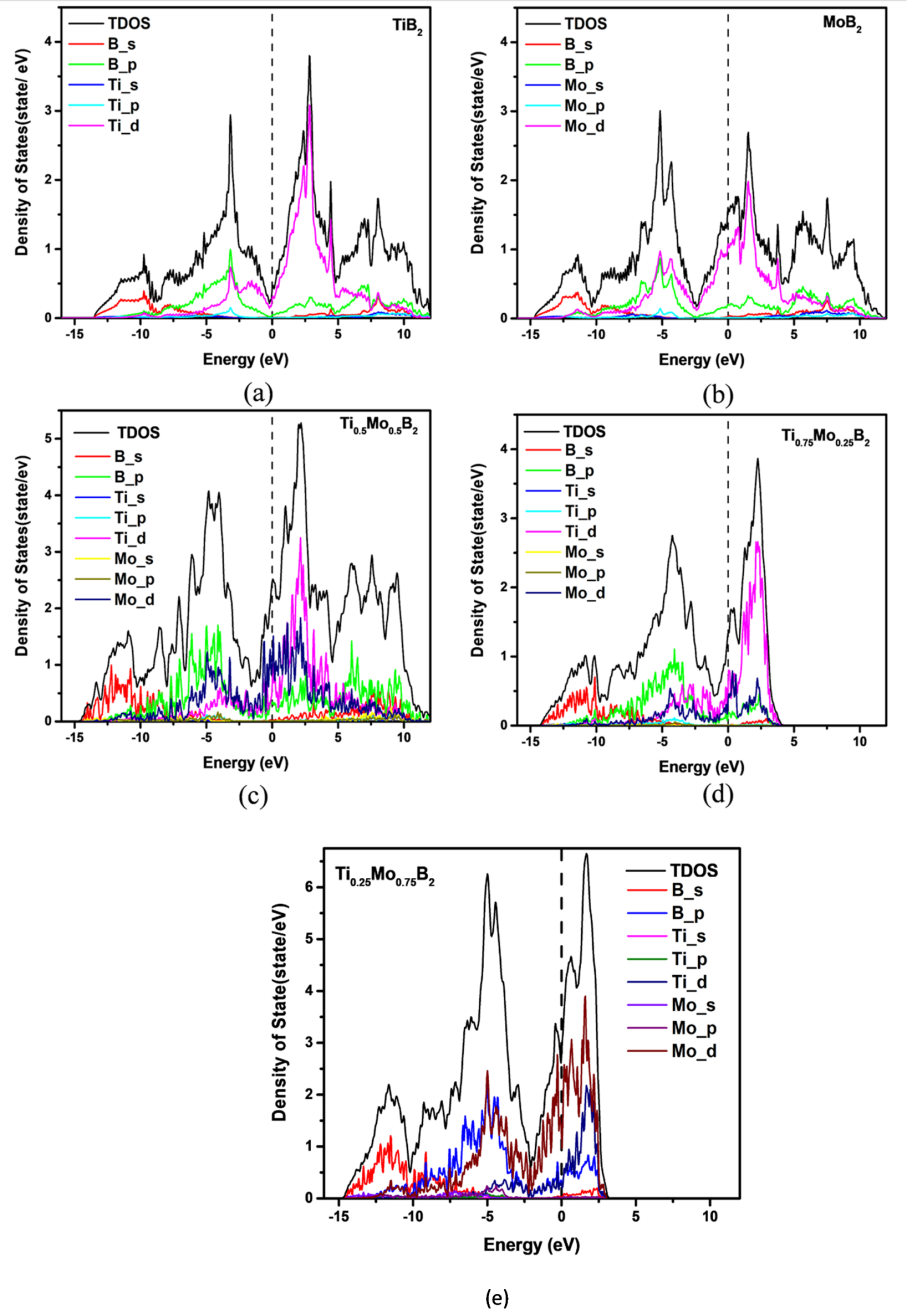
The calculated projected density of state (**a**) TiB_2_ (**b**) MoB_2_ (**c**) Ti_0.5_Mo_0.5_B_2_ (**d**) Ti_0.75_Mo_0.25_B_2_ and (**e**) Ti_0.25_Mo_0.75_B_2_.

Figure 3 illustrates the projected density of states (PDOS) of TiB_2_, MoB_2_, Ti_0.5_Mo_0.5_B_2_, Ti_0.75_Mo_0.25_B_2_, and Ti_0.25_Mo._075_B_2_, highlighting their bonding characteristics. In TiB_2_ (Fig. 3a) shows a prominent pseudogap at the Fermi level, indicating hybrid covalent-metallic bonding stabilized by strong Ti 3d and B 2p hybridization, reflecting a mix of covalent, metallic, and ionic interactions. For MoB_2_ (Fig. 3b), the pseudogap shifts to a lower energy level, suggesting weaker Mo 4d-B 2p covalent interactions compared to the stronger Ti 3d-B 2p bonding in TiB_2_. MoB_2_3 also exhibits a higher density of delocalized Mo 4d states near the Fermi level, indicating a more metallic character. Ti_0.5_Mo_0.5_B_2_ (Fig. 3c) combines the features of both parent compounds, with Ti 3d, Mo 4d, and B 2p states contributing to a uniform state distribution near the Fermi level. The shifted pseudogap at lower energy levels reflects a mixed bonding environment, demonstrating the hybrid electronic structure of the alloy with contributions from both covalent and metallic interactions.

For Ti_0.75_Mo_0.25_B_2_ (Fig. 3d), the projected density of states (PDOS) retains a TiB_2_-like character, where Ti 3d and B 2p orbitals dominate the bonding interactions. A noticeable pseudogap appears just below the Fermi level, indicating a slight shift compared to pure TiB_2_. The incorporation of Mo introduces additional 4d states, slightly increasing metallicity, but the strong Ti-B covalent interactions remain the primary bonding feature.

In Ti_0.25_Mo_0.75_B_2_ (Fig. 3e), the PDOS trends more toward MoB_2_-like behavior, with a significant contribution from Mo 4d states near the Fermi level. The pseudogap shifts further, suggesting an enhanced metallic character due to Mo substitution. Despite this shift, residual Ti 3d interactions persist, maintaining some degree of covalent bonding with B. This results in a hybrid electronic environment, balancing metallicity with covalent stabilization.

**Fig. 4:**
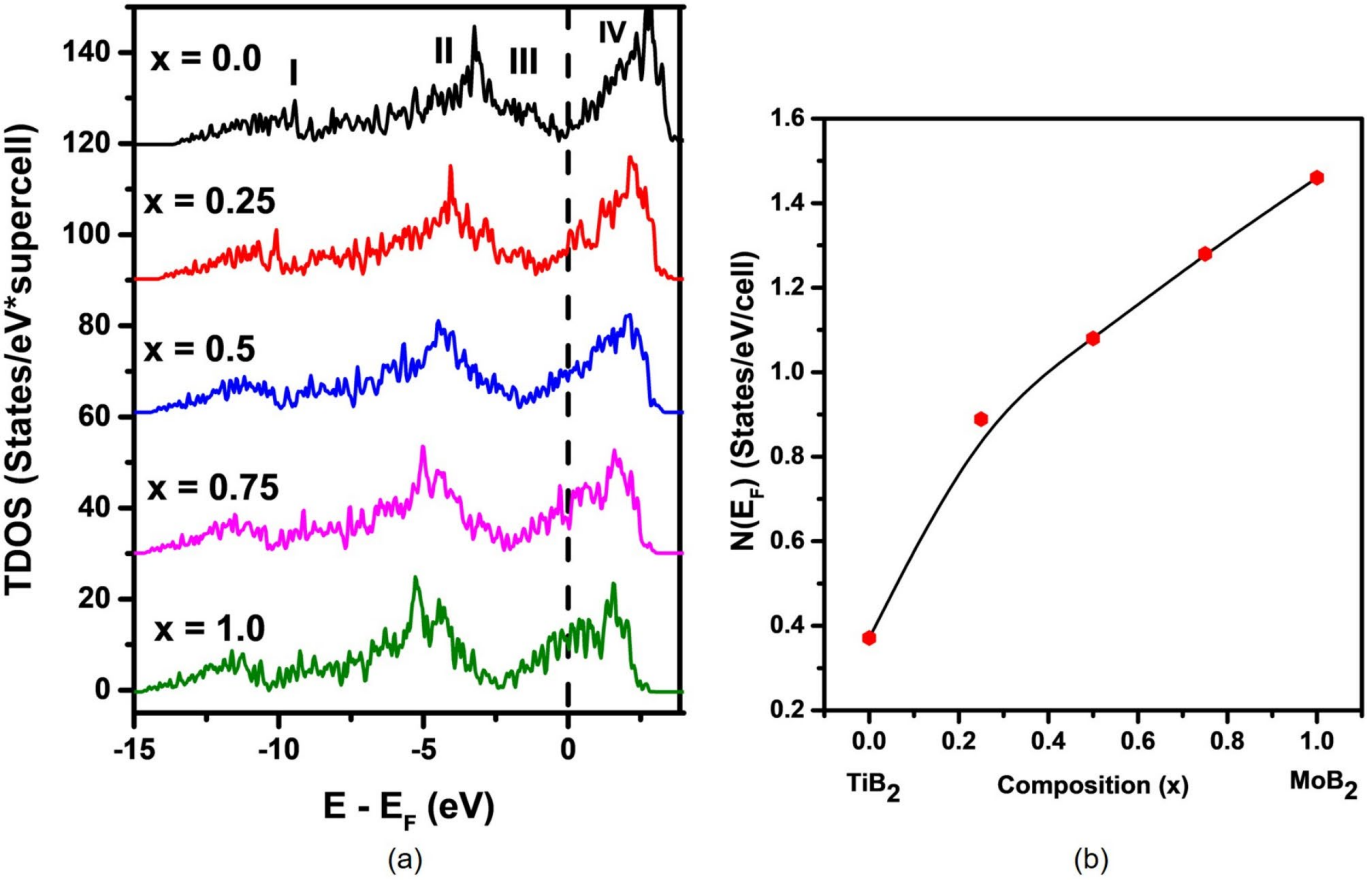
(**a**) Total density of states (TDOS) for Ti_1−x_Mo_x_B_2_ alloys (the 24-atoms supercells); (**b**) Density of states at the Fermi level, N(E_F_) as a function of x.

Figure 4a shows four main electronic bands labeled with Roman numerals. Band I primarily involve B 2s and metal d orbitals, while Band II arises from hybridized metal 3d and B 2p states. Just below the DOS minimum (pseudogap), an additional band beneath Band III is dominated by metal 3d orbitals. Band IV, above the Fermi level (E_F_), is primarily metal 3d states with some B 2p contributions. Transitioning from TiB_2_ to MoB_2_ shifts the Fermi level deeper into Band IV, reflecting MoB_2_’s enhanced metallic character.

Figure 4b represents the density of states at the Fermi level, N(E_F_), which signifies the number of electronic states per unit energy available at E_F_ a crucial factor influencing electrical conductivity and other electronic properties. Our calculation has pointed towards a linear increase in N(E_F_) from TiB_2_ to MoB_2_ with the increasing contribution of Mo d-electrons. Besides reflecting an increase in metallic character with increasing Mo content, it implies greater availability of electronic states for conduction. For the case of MoB_2_, the greater N(E_F_) is an expression of improved electrical conductivity, as a larger number of electrons would participate in conduction, thereby contributing to the enhanced metallic properties of the material.

**Fig. 5:**
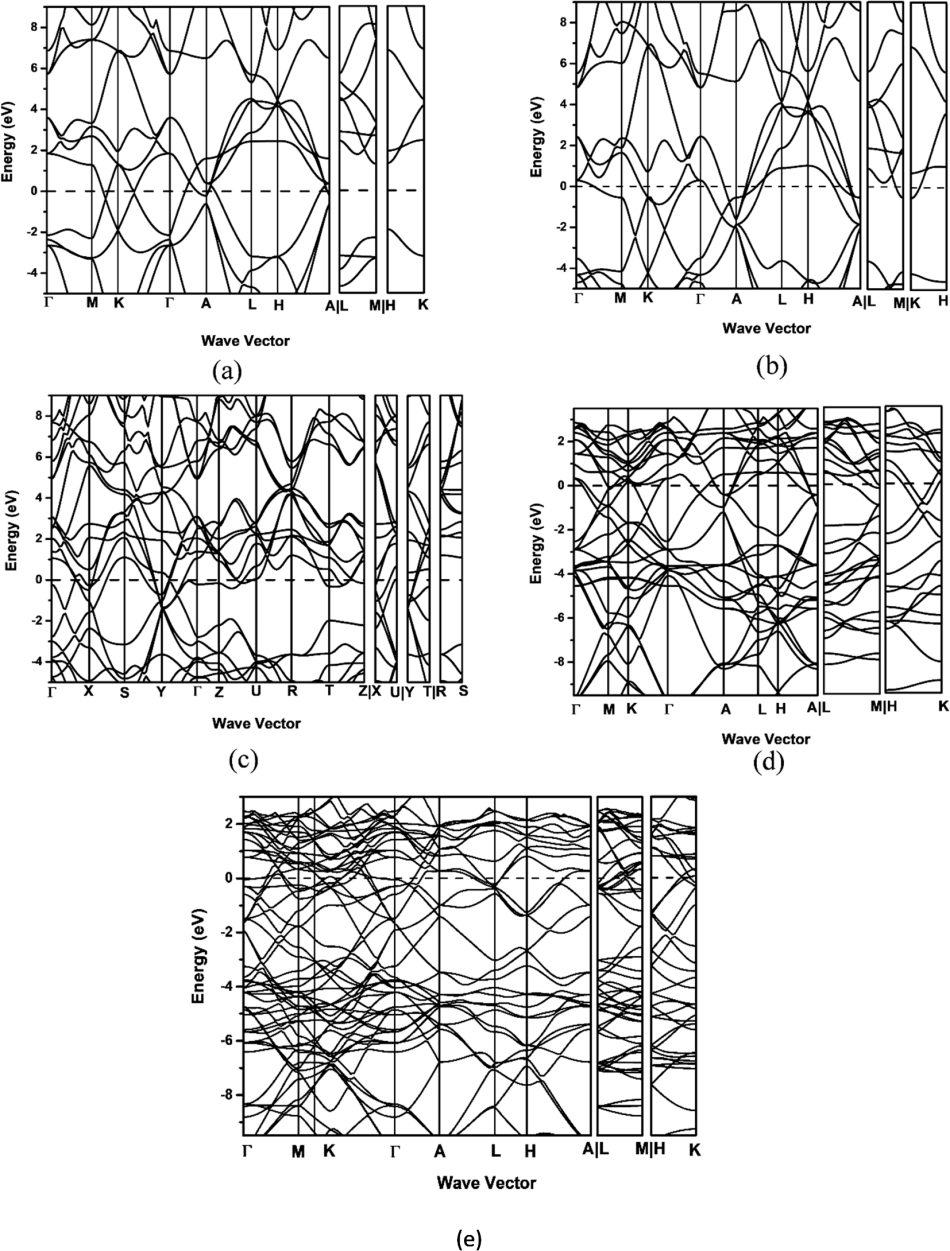
The calculated electronic band structure of (**a**) TiB_2_ (**b**) MoB_2_ and (**c**) Ti_0.5_Mo_0.5_B_2_ (The horizontal dashed line locates the Fermi energy (E_F_)).

The electronic band structure of TiB_2_ (Fig. 5a) shows metallic character with a high DOS at the Fermi level. Semi-metallic behavior at the Γ point and Dirac points at M − K and K − Γ confirm this classification^22–24^. The minimal band gap ensures excellent conductivity, though reduced band dispersion at the second Γ point limits charge carrier mobility compared to MoB_2_.

In contrast, MoB_2_ (Fig. 5b) displays stronger metallic behavior with higher DOS at the Fermi level and better charge transport due to its conduction band proximity to the Fermi level. Overlapping valence and conduction bands and greater band dispersion enhance charge carrier mobility, making it ideal for high-conductivity applications like electrodes. Ti_0.5_Mo_0.5_B_2_ (Fig. 5c) combines features of both, with alloying-induced shifts in VBM and CBM optimizing electronic interactions. Its hybrid band structure enhances mechanical stability, thermal resistance, and conductivity, making it suitable for advanced applications.

For Ti_0.75_Mo_0.25_B_2_ (Fig. 5d), the band structure retains features of TiB_2_, with metallic behavior and slight band broadening due to Mo incorporation. The increased dispersion of conduction bands suggests improved charge transport compared to pure TiB_2_. In Ti_0.25_Mo_0.75_B_2_ (Fig. 5e), the band structure becomes more MoB_2_-like, with higher conduction band occupation near the Fermi level. Enhanced band dispersion indicates greater charge carrier mobility, reflecting a transition toward stronger metallic behavior while still retaining some Ti-derived electronic states.

**Fig. 6:**
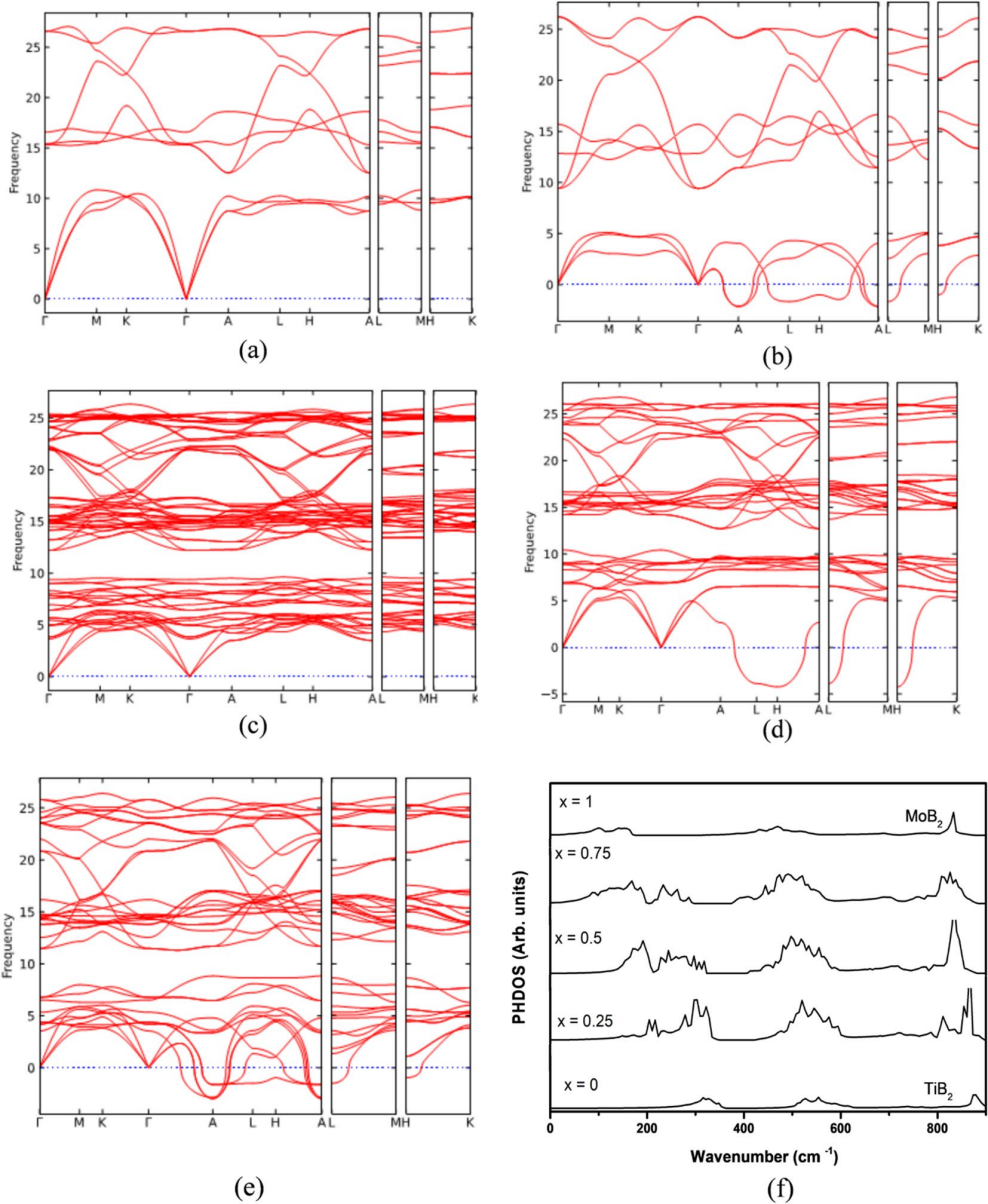
The calculated Phonon band structure of (**a**) TiB_2_ (**b**) MoB_2_ (**c**) Ti_0.5_Mo_0.5_B_2_ (**d**) Ti_0.75_Mo_0.25_B_2_ and (**e**) Ti_0.25_Mo_0.75_B_2_ (**f**) Calculated phonon density of states (PHDOS) for Ti_1−x_Mo_x_B_2_.

Phonon dispersion calculations provide insight into the dynamical stability of the Ti–Mo–B_2_ system. As shown in Fig. 6, the phonon band structure of pure TiB_2_ (Fig. 6a) exhibits no negative frequencies, confirming its dynamical stability. In contrast, MoB_2_ (Fig. 6b) shows negative frequencies, particularly near the Γ and A points, indicating dynamical instability. The Ti_0.5_Mo_0.5_B_2_ solid solution (Fig. 6c) is dynamically stable, as no imaginary modes appear in the phonon spectrum. However, Ti_0.25_Mo_0.75_B_2_ (Fig. 6e) and Ti_0.75_Mo_0.25_B_2_ (Fig. 6d) exhibit phonon instabilities, particularly near the A and L points, suggesting that these compositions may undergo structural distortions at low temperatures. The presence of negative frequencies in Mo-rich and Ti-rich compositions implies that an intermediate Ti/Mo ratio (x ≈ 0.5) favors structural stability, potentially due to optimized bonding interactions between Ti, Mo, and B atoms. These findings suggest that Ti_0.5_Mo_0.5_B_2_ is a promising composition for enhanced mechanical and thermal stability, while compositions deviating significantly from x = 0.5 may require further analysis of anharmonic effects or temperature-dependent stabilization mechanisms.

The PHDOS spectra (Fig. 6f) for Ti_1−x_Mo_x_B_2_ alloys show a slight shift in phonon modes toward lower wavenumbers as Mo content (x) increases. For x = 0 (pure TiB_2_), distinct peaks appear, particularly in the high-wavenumber region (~ 900 cm⁻¹). As Mo is introduced (x = 0.25 to x = 0.75), the spectra exhibit peak broadening and redistribution, indicating modifications in vibrational characteristics due to Mo incorporation. At x = 1 (pure MoB_2_), the high-wavenumber peaks remain noticeable but shift slightly to lower wavenumbers compared to TiB_2_. The overall phonon distribution also becomes smoother, suggesting a change in lattice dynamics. This trend implies that Mo substitution subtly alters the bonding environment and phonon dispersion, which may impact the alloy’s thermal and mechanical properties.

In addition to the Bader charge and band structure analysis for Ti_1–x_Mo_x_B_2_, the Electron Localization Function (ELF) was employed to explore electron localization and bonding nature. The ELF measures the likelihood of finding an electron near another with the same spin, providing insights into covalent and metallic bonding. It highlights regions of high electron localization, offering a deeper understanding of bonding interactions and electron correlation within these materials^25–27^.

**Fig. 7:**
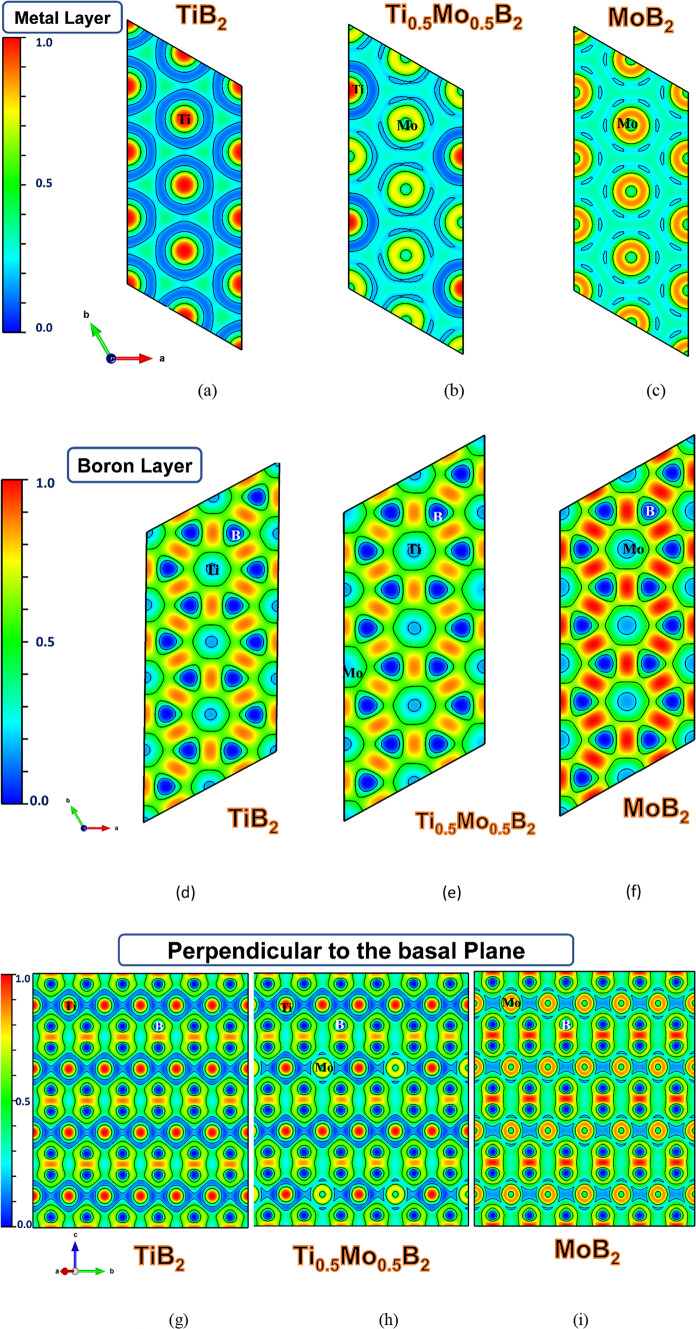
The electron localization function (ELF) of TiB_2_, Ti_0.5_Mo_0.5_B_2_, and MoB_2_, (**a**–**c**) the Metal Layers, (**d**–**f**) the Boron Layers, (**g**–**i**) the perpendicular layer to the basal plane.

Figure 7a–c illustrate the ELF analysis within the metal layers, parallel to the (0001) basal planes. A notable observation is the contrast in electron density behavior between TiB_2_, MoB_2_, and Ti_0.5_Mo_0.5_B_2_. In TiB_2_, the electron density is more localized around the titanium atoms, indicating stronger electron interactions and a higher degree of covalent character in the Ti-Ti bonds. In contrast, the electron density in MoB_2_ is more delocalized, signifying weaker covalent bonding and a higher degree of metallic character in the Mo-Mo bonds. This delocalization reflects the greater mobility of electrons in MoB_2_, contributing to its more metallic nature. The alloy, Ti_0.5_Mo_0.5_B_2_, presents an intermediate case, where the electron density shows a mix of localization and delocalization. This suggests that Ti_0.5_Mo_0.5_B_2_ combines characteristics from both parent compounds, with bonding and electron distribution behavior falling between the covalent nature of TiB_2_ and the metallic nature of MoB_2_.

Figure 7d–f show ELF analysis of the boron layers aligned with the (0001) basal planes in TiB_2_, Ti_0.5_Mo_0.5_B_2_, and MoB_2_, revealing strong covalent bonding. The ELF values indicate significant electron localization at the center of B-B bonds, forming a robust two-dimensional network in all three materials. While the covalent bonding strengths within the boron layers are similar, their integration into the crystal lattice varies significantly across the materials.

The ELF analysis perpendicular to the basal plane (i.e., along the c-axis), shown in Fig. 7g–i, further explains the metal-boron bonding characteristics. The Ti-B bonds in TiB_2_ exhibit moderate covalent bonding with some ionic character, while the Mo-B bonds in MoB_2_ display slightly weaker covalent interactions, indicating a greater ionic and metallic character. This finding suggests that Mo-B bonds are less covalent than Ti-B bonds, which aligns with the reduced interlayer connectivity observed in MoB_2_.

TiB_2_ demonstrates a more effective integration of the boron network with titanium atoms, contributing to its superior stiffness and hardness compared to MoB_2_ (As shown in mechanical propreties Section). And also TiB_2_ has been experimentally confirmed to be harder than MoB_2_, a result attributed to its strong covalent bonding and higher electron concentration. While the bulk modulus is related to resistance against compressibility, the hardness of MoB_2_ rather depends on electron concentration than bulk structural attributes^28,29^. Higher bulk modulus in MoB_2_ compared to TiB_2_ may be explained by a larger atomic size of molybdenum and increased atomic packing density causing greater resistance to compression. However, this electron density in TiB_2_, mainly in the Ti-B bonds, gives rise to stronger interlayer interactions and more robust covalent bonding. These enhanced electron localizations in TiB_2_ let it have shorter and hence more resilient bonds, which explains a higher value of hardness within the lower bulk modulus. These stronger interlayer interactions contribute further to enhanced elastic moduli, reduced slip systems which turn TiB_2_ into one of the ultra-high-performance materials with respect to mechanical properties.

### Elastic and mechanical properties of the alloy

The elastic constants of the TiB_2_–MoB_2_ alloys, as outlined in Table 2, offer critical insights into how Mo substitution affects mechanical behavior and structural stability. In hexagonal crystals like TiB_2_, there are five independent elastic constants (C_11_, C_12_, C_13_, C_33_, and C_44_) and the Born–Huang stability criteria must be met to ensure mechanical stability. These criteria for hexagonal systems are^36^:

2 $$\:{C}_{33}>\:0,\:{C}_{44}\:>\:0,\:{C}_{11}>\:\left|{C}_{12}\right|,\:\left({C}_{11}+{C}_{12}\right){\:C}_{33}>\:2{C}_{13}^{2}$$

**Table 2:** Calculated and experimental elastic constants Cij (in GPa) polycrystalline data, demonstrating the effect of mo substitution on elastic constants.

Sample	C_11_​	C_12_	C_13_​	C_33​_	C_44_​	Reference
TiB_2_	650.7 ± 2.92	70.1 ± 0.30	102.7 ± 2.74	451.9 ± 2.24	257.8 ± 2.08	This work
	654.5	56.5	98.4	454.5	263.2	Experimental^30^
	660	48	93	432	260	Experimental^31^
	656	66	98	461	259	Theoretical^32^
	650. 6	68.5	102.1	461.3	291.1	Theoretical^33^
Ti_0.75_Mo_0.25_B_2_	637.1 ± 3.25	90.3 ± 3.24	133.2 ± 2.97	433.4 ± 3.14	237.8 ± 0.26	This work
Ti_0.5_Mo_0.5_B_2_	624.6 ± 2.28	118 ± 2.71	154 ± 0.84	453.8 ± 1.08	208.1 ± 2.56	This work
Ti_0.25_Mo_0.75_B_2_	611.3 ± 3.65	113 ± 3.86	215.1 ± 2.88	381.7 ± 3.18	186 ± 3.87	This work
α-MoB_2_	623.9 ± 2.35	129 ± 1.40	231.5 ± 2.41	424 ± 3.75	164 ± 2.85	This work
	613	120	220	391	168	Experimental^29^
	609.2	149.5	196.3	393.3	155.5	Theoretical^34^
	627.0	120.0	231.0	398.0	174.0	Theoretical^35^

All TiB_2_-MoB_2_ supercells meet these criteria, confirming their mechanical stability.

For pure TiB_2_, the highest values of C_11_ (650.7 GPa) and C_33_ (451.9 GPa) reflect its exceptional rigidity and directional bonding strength, particularly along the a-axis (C_11_) and c-axis (C_33_). This strong resistance to deformation, especially along the c-axis, aligns with TiB_2_’s known stiffness and structural integrity. These values are consistent with both experimental and theoretical data^30–33^.

As Mo is introduced, a general trend of reduced longitudinal moduli C_11_ and C_33_ is observed. For Ti_0.75_Mo_0.25_B_2_, C_11_ drops to 637.1 GPa, while C_33_ decreases slightly to 433.4 GPa. In Ti_0.5_Mo_0.5_B_2_, however, C_33_ increases marginally to 453.8 GPa, suggesting a non-linear response in axial stiffness as Mo content rises. The trend for C_33_, while predominantly decreasing across the samples, exhibits less linearity compared to the more consistent reduction in C_11_. This irregularity suggests that the interaction of Mo atoms with the hexagonal lattice, particularly along the c-axis, is more complex, possibly due to changes in bonding character. As the Mo content increases further in Ti_0.25_Mo_0.75_B_2_ and pure MoB_2_, C_33_ reduces more significantly, reaching 381.7 GPa and 424.0 GPa, respectively. This marks a clear softening of axial stiffness at higher Mo concentrations.

In contrast to the decreasing trend in longitudinal moduli, the normal stress-related constants C₁₂ and C₁₃ exhibit a systematic increase with Mo substitution. For instance, C₁₃ increases from 102.7 GPa in TiB_2_ to 231.5 GPa in pure MoB_2_, indicating enhanced resistance to deformation under normal stresses in directions perpendicular to the a-axis and c-axis. This consistent trend across all alloys suggests that Mo substitution increases the material’s capacity to resist normal stress deformation, particularly in off-axial directions. However, the shear-related elastic constant C₄₄ shows a steady decrease as the Mo content increases, declining from 257.8 GPa in TiB_2_ to 164.0 GPa in MoB_2_. This suggests a reduction in the material’s ability to resist shear deformation, particularly in planes perpendicular to the c-axis.

Overall, Mo substitution in TiB_2_ leads to a reduction in longitudinal stiffness particularly in C₁₁ and C₃₃, with C₃₃ showing a less linear trend while simultaneously enhancing resistance to normal stresses, as indicated by the increase in C₁₂ and C₁₃. Despite the observed softening of certain elastic moduli, all supercells remain mechanically stable, making Ti–Mo–B_2_ alloys promising candidates for applications requiring both stiffness and enhanced normal stress resistance^29,34,35^.

**Fig. 8:**
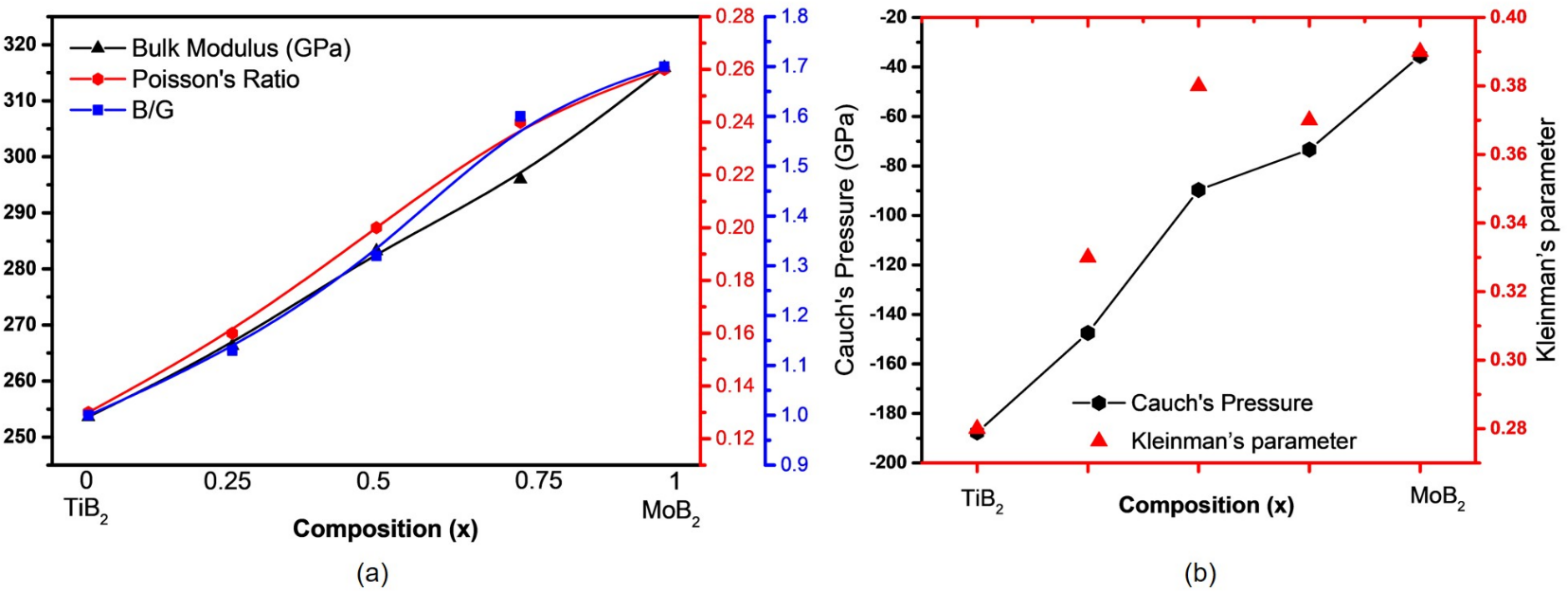
(**a**) The calculated Bulk modulus, Poisson’s and Pugh’s ratio. (**b**) Cauchy’s pressure and Kleinman’s parameters versus composition of Ti_1–x_Mo_x_B_2_.

Table 3 and reveal the impact of Mo on mechanical properties. The bulk modulus (B) increases linearly with Mo content, from 253.6 GPa (TiB_2_) to 315.9 GPa (MoB_2_), indicating enhanced volumetric deformation resistance due to Mo’s d-electron contributions (Fig. 8a). In contrast, Young’s modulus (E) and shear modulus (G) decrease from 576.0 GPa and 256.8 GPa for TiB_2_ to 449.9 GPa and 178.3 GPa for MoB_2_, reflecting reduced stiffness as Mo disrupts the rigid TiB_2_ bonding network.

**Table 3:** The calculated bulk modulus (B), Young’s modulus (E), shear modulus (G), Poisson’s ratio (ν), Pugh’s ratio (B/G), and hardness (Hv).

Sample	B	E	G	ν	B/G	Hv	Reference
TiB_2_	253.6 ± 1.53	576.0 ± 2.19	256.8 ± 0.81	0.13 ± 0.01	1.00 ± 0.02	37.9 ± 0.12	This Work
	250.0	583.5	262.6	0.11		31.9	Experimental^30,37^
	256.4	586.8	259.7	0.12		35.7	Theoretical^16,33^
Ti_0.75_Mo_0.25_B_2_	266.3 ± 2.01	546.9 ± 3.44	236.2 ± 3.39	0.16 ± 0.01	1.13 ± 0.01	34.8 ± 0.50	This Work
Ti_0.5_Mo_0.5_B_2_	283.2 ± 3.39	515.5 ± 2.38	215.3 ± 3.40	0.20 ± 0.01	1.32 ± 0.05	31.8 ± 0.50	This Work
Ti_0.25_Mo_0.75_B_2_	296.0 ± 0.24	459.4 ± 2.25	185.1 ± 2.72	0.24 ± 0.00	1.60 ± 0.02	27.3 ± 0.4	This Work
MoB_2_	315.9 ± 3.24	449.9 ± 1.47	178.3 ± 1.95	0.26 ± 0.01	1.70 ± 1.48	26.3 ± 0.29	This Work
	304	463	186			15.2	Experimental^29^

All the moduli are reported in GPa, except for the dimensionless values of B/G and ν.

The Cauchy pressure, a key indicator of atomic bonding, was calculated as C_12_-C_44_ using VASPKIT providing a single value to assess the bonding nature of Ti–Mo–B_2_ alloys. Negative values denote covalent bonding, while positive values suggest metallic bonding and increased ductility^38^. As shown in Fig. 8b, all calculated pressures are negative, highlighting the predominance of covalent interactions. TiB_2_ exhibits the most negative Cauchy pressure (-187.7 GPa), indicating strong covalent bonds and high brittleness. With increasing Mo content, the pressure becomes less negative, signaling a gradual shift toward metallic behavior, with MoB_2_ showing significantly reduced covalent bonding (-35.5 GPa).

The Kleinman parameters (0.28–0.39) further indicate bond-stretching behavior (ζ < 0.5), with ductility increasing as Mo content rises^39^. This systematic variation in Cauchy pressure and Kleinman parameters demonstrates the potential to tailor mechanical properties by adjusting the Ti-Mo ratio, balancing strength and ductility.

As shown in Fig. 8a Poisson’s ratio (ν) and Pugh’s ratio (B/G) also increase, signifying improved ductility with Mo incorporation, while hardness decreases from 37.9 GPa (TiB_2_) to 26.3 GPa (MoB_2_), indicating a trade-off between ductility and wear resistance. This trend demonstrates how Mo-rich alloys sacrifice hardness for improved flexibility. These findings align with existing theoretical and experimental results and highlight opportunities to optimize mechanical properties for specific applications^29,40^.

### Thermal properties: Debye temperature and melting point

As the data provided in Table 4, the thermal properties of the TiB_2_-MoB_2_ composites were probed by calculating the Debye temperature and estimating the melting point based on elastic constants. The Debye temperature, representing the material’s vibrational properties, thermal conductivity, and lattice stiffness, was calculated by VASPKIT^41^. In fact, the code obtains the Debye temperature from the sound velocities of the material-longitudinal and transverse acoustic wave velocities-which, in turn, are computed with the use of elastic constants and density.

**Table 4:** Calculated Debye and melting temperatures (in K) for the TiB_2_–MoB_2_ alloys.

	Sample	Debye temperature (K)	Melting temperature (K)
1	TiB_2_	1204 ± 1	$\:2984.2\pm\:300\:$
2	Ti_0.75_Mo_0.25_B_2_	1072 ± 2	$\:2915.5\pm\:300$
3	Ti_0.5_Mo_0.5_B_2_	963 ± 2	$\:2908.4\pm\:300$
4	Ti_0.25_Mo_0.75_B_2_	835 ± 2	$\:2760.5\pm\:300$
5	MoB_2_	789 ± 1	$\:2861.8\pm\:300$

The Debye temperature ($\:{{\theta}}_{D}$) was computed using the following formula:

3 $$\:{{\theta}}_{D}=\frac{h}{{k}_{B}}{\left[\frac{3n}{4\pi\:}\frac{N\rho\:}{M}\right]}^{\frac{1}{3}}{v}_{m}$$

Where k_B_ is the Boltzmann’s constant, h is the Planck’s constant, is the number of atoms in the cell, N is the Avogadro’s number, M is the molecular weight and $\:\rho\:$ is the density of material and $\:{v}_{m}$ is the average sound velocity, which depends on the longitudinal and transverse sound velocities.

To estimate the melting temperature^42,43^, the following empirical formula was applied:

4 $$T_{m} = 354 K + 1.5^{\:K}/_{GPa}\times\left( {2C_{{11}} + C_{{33}} } \right) \pm 300 K$$

This formula approximates the melting point based on the material’s stiffness, as indicated by the longitudinal elastic constants (C₁₁ and C₃₃). The ± 300 K uncertainty means the estimates are approximate rather than precise.

The crystalline material’s melting points are generally related to their bonding energy and thermal expansion. In general, the higher the melting point, the lower the thermal expansion, along with high cohesive energy because of strong atomic interaction^42^. It is also further related in view of predicting temperature at which materials could remain functional without considerable distortion, chemical alteration, and oxidation.

The calculated data (Table 4) reveals a pronounced decrease in both Debye and melting temperatures as Mo content increases in the TiB_2_-MoB_2_ alloy system. The significant reduction in the Debye temperature reflects the gradual softening of the lattice, indicating weaker atomic bonding as Mo replaces Ti. This trend can be attributed to Mo’s lower bond strength and larger atomic radius, which disrupts the stiffness of the lattice structure.

The corresponding decline in melting temperatures further underscores this weakening effect. As Mo concentration rises, the reduced atomic bond strength leads to a lower energy requirement for breaking the lattice, thereby lowering the melting point. This pattern is consistent with the physical properties of the individual borides: TiB_2_ shows superior lattice stability and higher hardness due to its stronger bonding, while MoB_2_ is softer.

Despite some variation between the calculated melting points and experimentally reported values, for pure TiB_2_ and MoB_2_, the estimates generally fall within a reasonable ± 300 K error margin. For instance, the experimental melting points of TiB_2_ and MoB_2_ are close to the upper and lower bounds of the computational predictions, suggesting the calculated values provide a reliable approximation. This agreement validates the empirical models used and positions these results as a useful foundation for predicting melting points of unknown alloys in this system. These findings also open pathways for further experimental studies and advanced simulations to fine-tune predictions and explore their applicability in high-performance materials.

## Conclusions

The detailed study of the structural, electronic, thermal and mechanical properties of Ti_1−x_Mo_x_B_2_ alloys has been performed using first-principles DFT calculations. The negative mixing energies confirm alloy stability across all compositions, supporting the formation of continuous solid solutions. Phonon dispersion calculations show that the Ti_0.5_Mo_0.5_B_2_ solid solution exhibits dynamical stability, making it a promising composition for enhanced mechanical and thermal stability, while Ti- and Mo-rich compositions may require further investigation. Deviations from Vegard’s Law, combined with increased lattice volume due to Mo substitution, suggest lattice distortions that are stabilized by Ti-Mo bonding. In terms of mechanical properties, increasing Mo content enhances ductility, as indicated by higher Poisson’s ratios and B/G ratios, while reducing stiffness and hardness. Electronically, the density of states at the Fermi level shows a linear increase from TiB_2_ to MoB_2_, driven by the rising contribution of Mo *d*-electrons, which enhances metallicity and increases available states for conduction. Thermal properties, such as Debye temperatures and melting points, decrease progressively with increasing Mo content, aligning with experimental trends. These findings provide a framework for designing Ti_1−x_Mo_x_B_2_ alloys with tailored mechanical and thermal properties, offering valuable insights for future experimental validation and high-performance engineering applications.

## Methods

First-principles calculations based on Density Functional Theory (DFT) were performed using the Vienna Ab-initio Simulation Package (VASP)^44–46^ with the projector augmented-wave (PAW) method^47^. The Perdew-Burke-Ernzerhof (PBE) generalized gradient approximation (GGA)^48^ was used for the exchange-correlation functional. PAW pseudopotentials for Ti, Mo, and B atoms were applied considering the following valence electron configurations: Ti (3d^2^4s^1^), Mo (4d^5^5s^1^), and B (2s^2^2p^1^), allowing for accurate simulation of interactions involving the core electrons at reasonable computational cost^49^.

A 2 × 2 × 2 hexagonal TiB_2_ supercell (space group P6/mmm) containing 24 atoms was modeled. Five alloy compositions (TiB_2_, Ti_0.75_Mo_0.25_B_2_, Ti_0.5_Mo_0.5_B_2_, Ti_0.25_Mo_0.75_B_2_, MoB_2_) were studied, with results averaged over three representative atomic configurations for each composition. Lattice parameters were optimized until forces were below 10^−4^ eV/Å, with total-energy convergence set to 10^−8^ eV. The saturation tests revealed that the required energy cutoff for the plane-wave basis should be 600 eV, while a Γ-centered Monkhorst-Pack k-point mesh with a spacing of 0.03 Å⁻¹ was used^50^. The pre- and post-processing, including input generation, were done by VASPKIT^41^.

Bader charge analysis^51,52^ was performed in order to explore charge transfer and bonding between Ti, Mo, and B atoms. The elastic constants (C_ij_) were computed by the energy-strain method. Minor strains were applied to the supercells, and related total energies were calculated^41,53–55^. To assess mechanical properties, bulk modulus (B), shear modulus (G), Young’s modulus (E), and Poisson’s ratio (ν) were calculated by using the Voigt-Reuss-Hill averaging scheme^41,56,57^. Furthermore, the theoretical Vickers hardness was estimated by using reference^58^. The melting temperatures also have been estimated using an empirical relationship taking into account longitudinal moduli of C₁₁ and C₃₃^42,43,59,60^. Crystal structures are visualized by VESTA^61^, allowing analysis of ELF.

## Data Availability

All the data has been revealed to readers in the manuscript.
